# Supine versus semi-Fowler’s positions for tracheal extubation in abdominal surgery-a randomized clinical trial

**DOI:** 10.1186/s12871-020-01108-5

**Published:** 2020-08-01

**Authors:** Qiongfang Zhu, Zheyan Huang, Qiaomei Ma, Zehui Wu, Yubo Kang, Miaoyin Zhang, Tiantian Gan, Minxue Wang, Fei Huang

**Affiliations:** 1 Department of Anesthesiology, The First Affiliated Hospital of Sun Yat-sen University, Guangzhou, China; 2 Department of Anesthesiology, The Third Affiliated Hospital of Sun Yat-sen University, 600 Tianhe Rd, Guangzhou, China

**Keywords:** Tracheal extubation; supine; semi-Fowler’s; post-anesthesia care unit

## Abstract

**Background:**

Tracheal extubation is commonly performed in the supine position. However, in patients undergoing abdominal surgery, the supine position increases abdominal wall tension, especially during coughing and deep breathing, which may aggravate pain and lead to abdominal wound dehiscence. The semi-Fowler’s position may reduce abdominal wall tension, but its safety and comfort in tracheal extubation have not been reported. We aimed to evaluate the safety and comfort of different extubation positions in patients undergoing abdominal surgery.

**Methods:**

We enrolled 141 patients with an American Society of Anesthesiologists grade of I-III who underwent abdominal surgery. All patients were anesthetized with propofol, fentanyl, cisatracurium, and sevoflurane. After surgery, all patients were transferred to the post-anesthesia care unit (PACU). Patients were then randomly put into the semi-Fowler’s (*n* = 70) or supine (*n* = 71) position while 100% oxygen was administered. The endotracheal tube was removed after the patients opened their eyes and regained consciousness. Vital signs, coughing, and pain and comfort scores before and/or after extubation were recorded until the patients left the PACU.

**Results:**

In comparison with the supine position, the semi-Fowler’s position significantly decreased the wound pain scores at all intervals after extubation (3.51 ± 2.50 vs. 4.58 ± 2.26, 2.23 ± 1.68 vs. 3.11 ± 2.00, 1.81 ± 1.32 vs. 2.59 ± 1.88, *P* = 0.009, 0.005 and 0.005, respectively), reduced severe coughing (8[11.43%] vs. 21[29.58%], *P* = 0.008) and bucking after extubation (3[4.29%] vs. 18[25.35%], *P* < 0.001), and improved the comfort scores 5 min after extubation (6.11 ± 2.30 vs. 5.17 ± 1.78, *P* = 0.007) and when leaving from post-anesthesia care unit (7.17 ± 2.27 vs. 6.44 ± 1.79, *P* = 0.034). The incidences of vomiting, emergence agitation, and respiratory complications were of no significant difference.

**Conclusion:**

Tracheal extubation in the semi-Fowler’s position is associated with less coughing, sputum suction, and pain, and more comfort, without specific adverse effects when compared to the conventional supine position.

**Trial registration:**

Chinese Clinical Trial Registry, ChiCTR1900025566. Registered on 1st September 2019.

## Background

The post-anesthesia care unit (PACU) provides close monitoring for postoperative patients who are not fully awake after general anesthesia. Due to the residual effects of anesthetics and muscle relaxants, admittance into the PACU is associated with a high risk of complications. A retrospective study of 18,473 patients found that the overall incidence of PACU complications was 23% and the most common complications included postoperative nausea and vomiting (10 to 30%), upper airway abnormalities (6.9%), hypotension (2.7%), arrhythmia (1.4%), hypertension (1.1%), and altered consciousness (0.6%) [1].

Studies have shown that the extubation position during emergence from anesthesia is related to the peri- and postoperative complications. For patients with obstructive sleep apnea after uvulopalatopharyngoplasty, extubation in the upright position can significantly reduce the upper airway blocking, the work of breathing, postoperative respiratory depression, and increase functional residual capacity [2] Another study found that extubation in the prone position can significantly reduce postoperative coughing in patients undergoing spinal surgery [3]. Because there is currently no evidence that a single extubation position is suitable for all patients, we assumed that patients should be placed in different positions based on their conditions.

Abdominal surgery patients have a high risk of postoperative nausea and vomiting [4]. After administering general anesthesia, most anesthetists prefer to place patients in the supine position for extubation. This is because it is simple, enables easy observation, and can prevent regurgitation in the case of vomiting [5]. However, some believe that awake extubation recovers protective reflexes, such as coughing and swallowing, after extubation; in this case, the advantages of supine extubation are diminished [6]. Besides, abdominal pain after surgery leads to respiratory restriction and increased abdominal wall pressure [7]. Postoperative coughing, which is helpful for sputum excretion and recovery of pulmonary function, yet further increases abdominal pressure and aggravates the pain, for which patients are more unwilling to cough actively in the supine position. A better position for extubation after abdominal surgery should be used postoperatively to achieve less abdominal pain and better patient comfort while not increasing the workload of paramedics in the PACU.

In the semi-Fowler’s position, the extension of abdominal muscles decreases, thereby potentially relieving the intension of surgical wound and abdominal pain. In addition, peritoneal effusion is restricted to the lower position, leading to a more adequate drainage. Moreover, studies have shown that the semi-Fowler’s position can increase the lung capacity by 10 to 15% and improve the range of motion of diaphragm muscle; this facilitates lung expansion and increases gas exchange [8]. One study revealed that using the semi-Fowler’s position within 24 h of tracheal intubation significantly reduced ventilator-associated pneumonia. In addition, early postural interventions after general anesthesia can facilitate pulmonary ventilation and increase blood oxygen content [9].

Therefore, we hypothesized that the semi-Fowler’s position might be more comfortable for emergence from anesthesia and extubation in patients undergoing abdominal surgery than the supine position, and reduce common complications in the PACU. To test this hypothesis, we conducted a prospective, randomized clinical trial and aimed to assess the comfort and safety in patients extubated in the semi-Fowler’s position compared to those extubated in the supine position.

## Methods

### Ethical considerations and trial registration

This study was approved by the Ethics Committee for Clinical Research and Animal Trials of the First Affiliated Hospital of Sun Yat-sen University (number [2019]225) on 29th May 2019. The study was registered on the Chinese Clinical Trial Registry (ChiCTR1800018537).URL:http://www.chictr.org.cn/showprojen.aspx?proj=42692

The study was conducted from 5th September 2019 through 17th February 2020 in the First Affiliated Hospital of Sun Yat-sen University. All enrolled patients signed informed consent before admission.

### Patients and sample size calculation

**Fig. 1:**
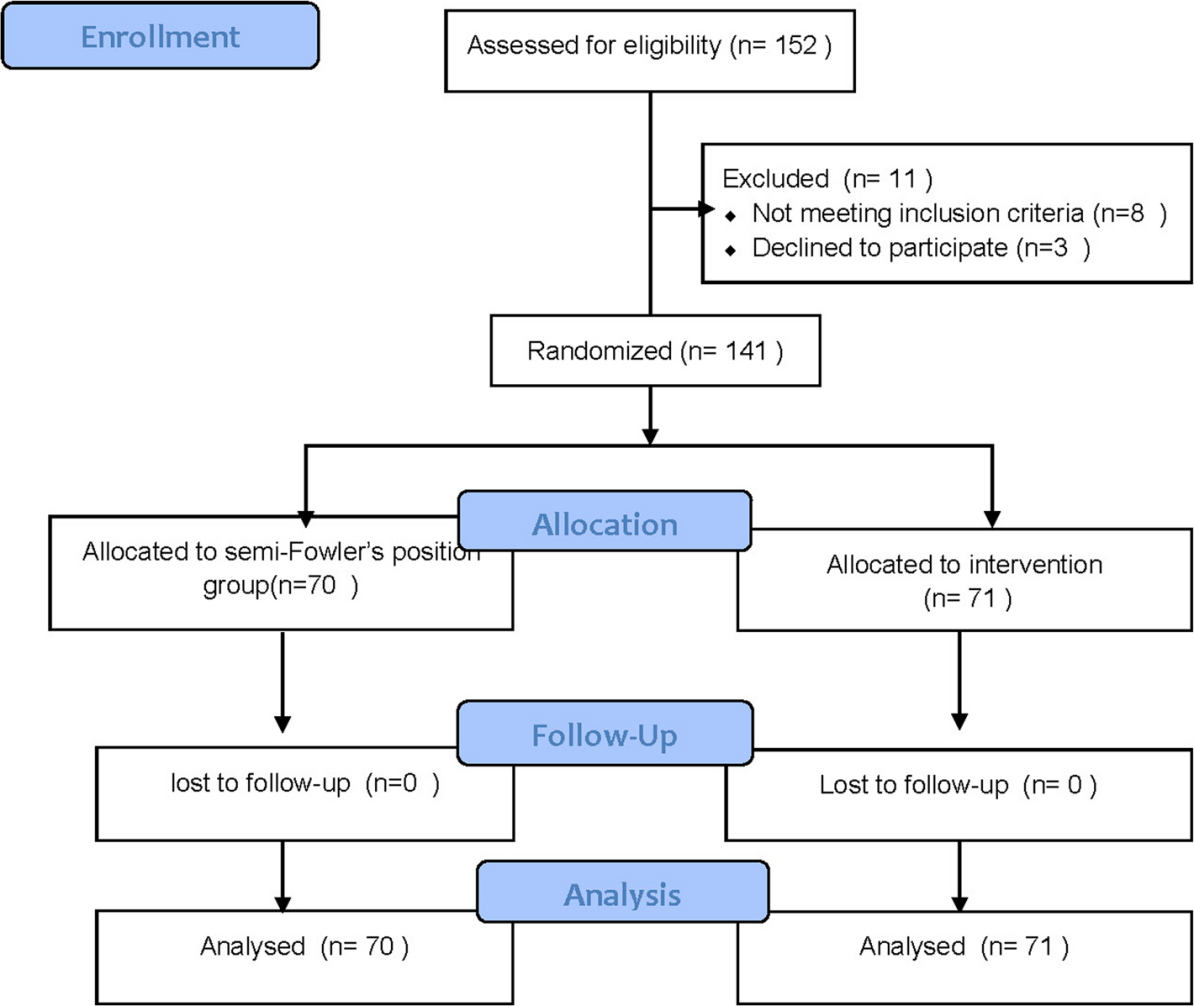
Participant flow diagram

A total of 152 patients aged 18–70 years were screened, of whom 141 were finally enrolled. All the patients were classified as American Society of Anesthesiologists (ASA) grade between I and III and were scheduled for laparoscopic or traditional open abdominal surgery under general anesthesia with endotracheal intubation. Patients with difficult airways, obesity (body mass index [BMI] > 35 kg/m^2^), or symptomatic reflux were excluded. Those who admitted to department of intensive care unit (ICU) were also excluded. The primary outcome of this study is the patient comfort visual analog scale (VAS) score 5 min after extubation. Based on the sample size formula with a two-sided alpha of 0.05 and a power of 0.8, an adequate sample size was determined to be 33 patients in each group. Accordingly, we recruited a sample of 141 patients for the study (Fig. 1).

### Group interventions

The patients enrolled were randomized (using a simple number table) and divided into the supine position group (control group, *n* = 71) or the semi-Fowler’s position group (experimental group, *n* = 70). All patients were blinded to their group assignment. Patients in the control group did not undergo a position change and received tracheal extubation in the supine position. In the experimental group, patients were placed in the semi-Fowler’s position (supine with 30° head-up) during emergence and extubation until they were discharged from the PACU. Patients in both groups were induced with midazolam, propofol, cisatracurium, and fentanyl and were anesthetized with sevoflurane.

### Study procedures and data collection

After enrollment, demographic data, including age, gender, body mass index (BMI), ASA class, NYHA class, Mallampati class, history of cigarette use, and breath holding test, were recorded.

The anesthetists in this study were not preselected and were given general guidelines to conduct the anesthetics. Baseline data, consisting of noninvasive mean arterial pressure (MAP), heart rate (HR), peripheral capillary oxygen saturation (SpO_2_), respiratory rate (RR), and temperature, were recorded before anesthesia (T0). Besides, type of surgery, estimated blood loss, crystalloid replacement, anesthesia time, and surgical time were recorded intraoperatively.

After the operation was completed and the drapes were removed, sevoflurane was discontinued and patients were given 100% oxygen instead. Patients were then transferred to the PACU, where they underwent standard electrocardiography and noninvasive blood pressure and peripheral capillary oxygen saturation monitoring. The patients were randomized to either the supine or semi-Fowler’s position and placed into corresponding position 5 min later. The patients had to achieve the following conditions before extubation: (1) spontaneous ventilation, (2) complete reversal of neuromuscular blockade, and (3) eye-opening and regaining of consciousness. Heart rate (HR), mean arterial pressure (MAP), and SpO2 were recorded at six points: (1) 5 min after arrival in PACU (T1), (2) immediately after positioning (T2), (3) the moment before extubation (T3), (4) 1 min after extubation (T4), (5) 5 min after extubation (T5), (6) 30 min after extubation (T6), and (7) when leaving the PACU (T7). Patients in both groups were suctioned before extubation, but were not stimulated in any other way until they could move spontaneously. Once extubation was carried out, extubation time (from arrival at PACU to extubation) was recorded. In addition to demographic data, the frequency of initiative severe coughing for sputum excretion (sustained ≥5 s), passive bucking due to stimulation of excretion, requirement for suction after extubation, vomiting, and emergence agitation, the Riker Sedation-Agitation Scale (SAS) score, airway rescue after extubation, the need for suctioning, sore throat, the wound pain VAS score, the Bruggemann comfort scale (BCS) score, the comfort VAS score, and satisfaction score from nursing personnel were recorded. The duration of PACU was also recorded.

Respiratory complications that occurred after extubation were recorded: (1) transient decline of SpO_2_ > 5% or SpO_2_ < 90% that yet requires no intervention; (2) upper airway obstruction or respiratory depression that needs noninvasive intervention(s), e.g., the jaw-thrust maneuver, the administration of oro−/naso-pharyngeal airway or noninvasive positive ventilation; (3) severe airway obstruction or respiratory depression that needs reintubation.

During the study, we became aware of the use of dexmedetomidine and lidocaine cream. To exclude the influence of these medicines on the results, we retrospectively collected data regarding their use.

### Statistical analyses

Patient demographic parameters, HR, and MAP were compared between the groups at baseline using the t-test with Satterthwaite adjustments for unequal variance, when appropriate. The normality of the distribution was assessed using the Shapiro-Wilk test. The parametric data were expressed as mean (± standard deviation [SD]), while the nonparametric data were expressed as median (interquartile range). The categorical data were described as frequency (proportion). Significance in the comparisons between the two groups was assessed using the chi-squared test for categorical variables and the Student’s t-test (for data following the normal distribution) or Mann–Whitney U test (for data following an abnormal distribution) for quantitative variables. Anesthesia time, surgical time, estimated blood loss, and crystalloid replacement were log-normal in distribution, and thus required log transformation before the t-tests. Demographic and baseline data were summarized as mean values ± SD, geometric means with 95% confidence intervals, medians and range, or frequencies. *P*<0.05 was considered statistically significant. All statistical analyses were conducted using SPSS version 23.0 (IBM Corp., Armonk, NY, USA).

## Results

From September 2019 to February 2020, 152 patients were screened, of whom 141 were recruited in this study.70 semi-Fowler’s patients and 71 supine patients were compared. As in Table 1, there were no statistically significant differences in demographic data between the two groups. The intraoperative phase characteristics are also shown in Table 1. There were no statistical differences in anesthesia time, surgical time, extubation time, and PACU duration between the two groups. Estimated blood loss and crystalloid replacement were similar between the groups.

**Table 1:** Demographics by group

Demographic	Semi-fowler’s position	Supine position
Gender (Male/Female)	41/29	41/30
Age (mean yr ± SD)	50.4(±12.4)	52.7(±10.8)
ASA Class		
I	16 (22.9%)	17 (23.9%)
II	49 (70.0%)	50 (70.4%)
III	5 (7.1%)	7 (9.9%)
BMI (mean kg/m2 ± SD)	22.5(±3.0)	23.1(±3.4)
Baseline HR (mean beats/min ± SD)	76.3(±11.9)	79.6(±13.2)
Baseline MAP (mean mmHg±SD)	95.7(±13.7)	97.0(±13.0)
Baseline T (IQR centi-degree)	36.2 (36.1–36.5)	36.2 (36.1–36.6)
Baseline RR (IQR rate/min)	17 (13–20)	16 (13–18)
Sp02		
≥ 96%	68 (97.1%)	70 (98.6%)
<96%	2 (2.9%)	1 (1.4%)
Breath holding test (IQR seconds)	37 (32–43)	35 (28–47)
NYHA		
I	60 (85.7%)	59 (83.1%)
II	9 (12.9%)	7 (16.9%)
III	1 (1.4%)	
Mallampati		
I	40 (57.1%)	38 (53.5%)
II	28 (40.0%)	31 (43.7%)
III	2 (2.9%)	2 (2.8%)
Cigarette use	12 (17.1%)	13 (18.3%)
Type of surgery		
Laparoscopic surgery	18 (25.71%)	18 (25.35%)
Traditional open surgery	52 (74.29%)	53 (74.65%)
Dexmedetomidine	54 (77.14%)	59 (83.10)
lidocaine cream	68 (97.14%)	70 (98.59%)
Anesthesia time (mean min ± SD)	319(±130)	315(±114)
Surgical time (min)	259(±125)	256(±112)
Extubation time (min)	38.00(±25.35)	36.52(±25.27)
Duration in PACU (min)	72.8(±27.9)	76.1(±24.6)
Estimated blood loss (ml)	289.90(±488.46)	387.42(±663.91)
Crystalloid replacement (ml)	1974.14(±909.89)	2077.75(±816.74)

Results are expressed as mean ± SD with corresponding 95% confidential interval or median with interquartile rage [25–75%].SpO2 Peripheral oxygen saturation

Although the BCS scores in both groups were comparable, the comfort VAS scores for the semi-Fowler’s position were higher than those for the supine position at T5 and T7 (Table 2, *P* = 0.007 and 0.034, respectively). The wound pain VAS scores of the semi-Fowler’s position group were significantly lower than those of the supine position group at all intervals after extubation (Table 2, *P* = 0.009, 0.005, and 0.005, respectively). Besides, extubation in the semi-Fowler’s position did not aggravate the sore throat compared with that in the supine position.

**Table 2:** Results of pain and comfort

Outcome	Semi-fowler’s	Supine	*P* value
BCS (mean ± SD)			
5 min after extubation	1.94 ± 1.13	1.68 ± 1.05	0.149
30 min after extubation	2.27 ± 0.96	2.13 ± 0.94	0.368
Before leaving PACU	2.39 ± 0.95	2.26 ± 0.88	0.408
Comfort VAS (mean ± SD)			
5 min after extubation	6.11 ± 2.30	5.17 ± 1.78	0.007
30 min after extubation	6.70 ± 2.14	6.23 ± 1.63	0.140
Before leaving PACU	7.17 ± 2.27	6.44 ± 1.79	0.034
Sore throat VAS (mean ± SD)			
5 min after extubation	2.20 ± 2.27	2.11 ± 1.98	0.808
30 min after extubation	1.60 ± 1.96	1.54 ± 1.76	0.836
Before leaving PACU	1.44 ± 1.81	1.31 ± 1.75	0.658
Wound pain VAS (mean ± SD)			
5 min after extubation	3.51 ± 2.50	4.58 ± 2.26	0.009
30 min after extubation	2.23 ± 1.68	3.11 ± 2.00	0.005
Before leaving PACU	1.81 ± 1.32	2.59 ± 1.88	0.005
Satisfaction score from nursing personnel			0.011
4 points	70 (100%)	65 (91.55%)	
3 points	0 (0%)	3 (4.22%)	
2 points	0 (0%)	3 (4.22%)	
1 points	0 (0%)	0 (0%)	

All *P*-values are calculated by Chi-square or Fischer’s exact test

**Fig. 2:**
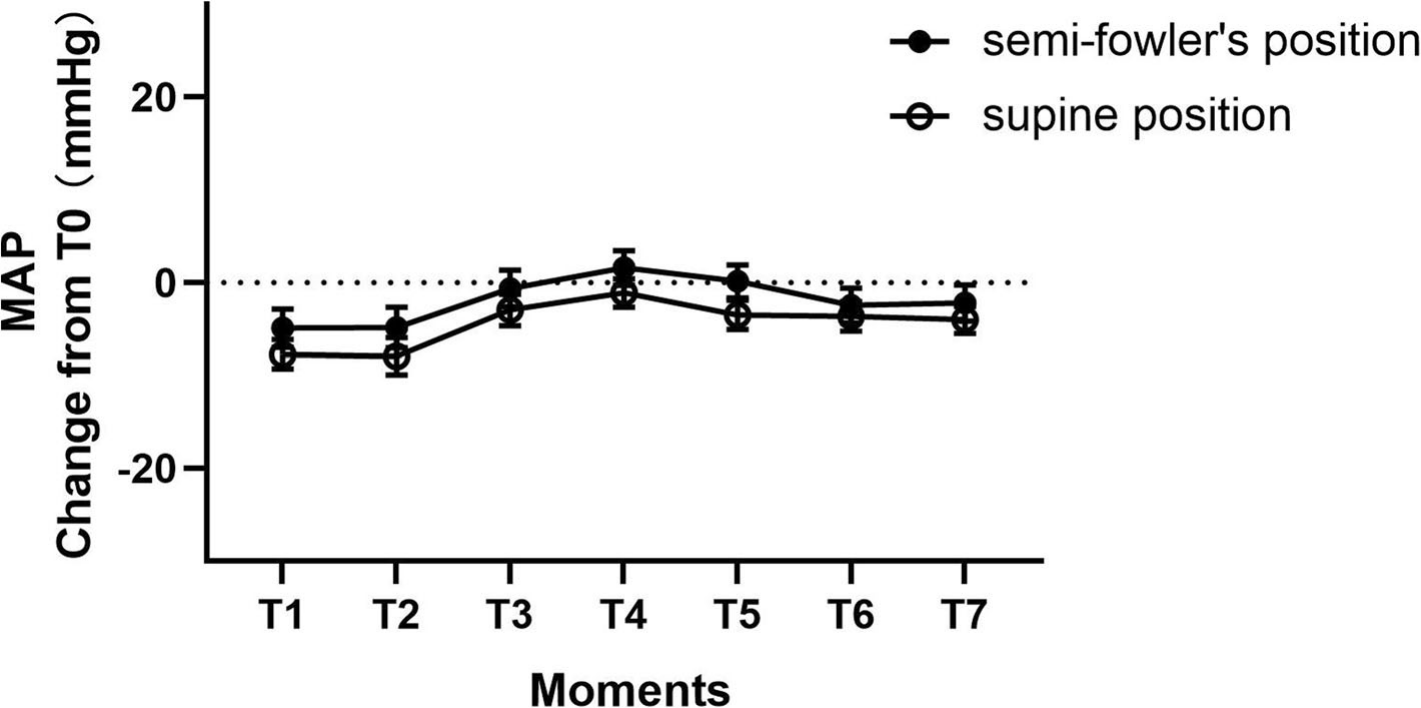
Mean change in mean arterial pressure (±SE) at each moments after the initiation of treatment. T1:5 min after transferred to PACU; T2:immediately after positioning; T3:the moment before extubation,; T4:1 min after extubation,; T5:5 min after extubation; T6:30 min after extubation,; T7: leaving the PACU

**Fig. 3:**
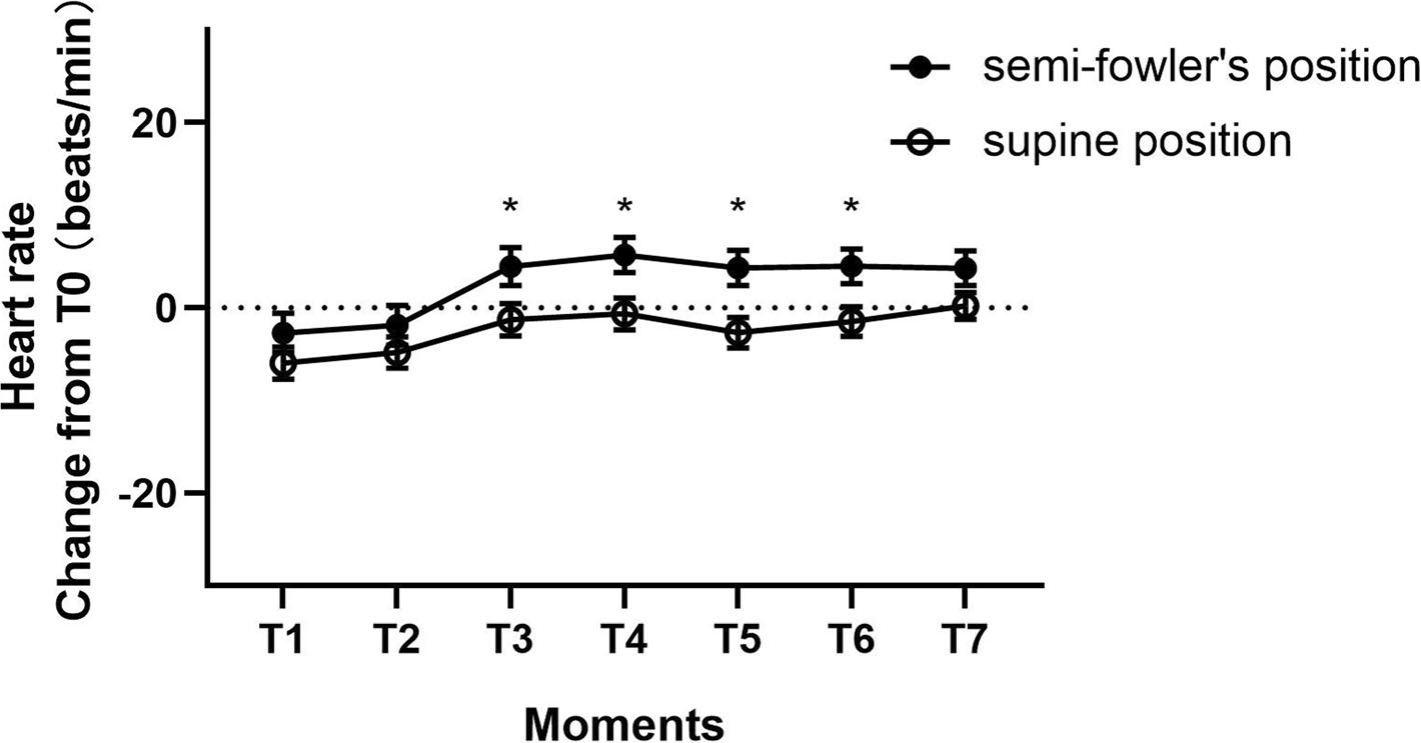
Mean change in heart rate from baseline (±SE) at each moments after the initiation of treatment. T1:5 min after transferred to PACU; T2:immediately after positioning; T3:the moment before extubation,; T4:1 min after extubation,; T5:5 min after extubation; T6:30 min after extubation,; T7: leaving the PACU

Patients in the semi-Fowler’s position had no statistically significant changes in respiratory rate, SpO_2_ or MAP from baseline at all intervals compared with patients in the supine position (Fig. 2). However, semi-Fowler’s patients demonstrated a statistically significant increase in HR, compared to baseline, at T3(*P* = 0.035), T4(*P* = 0.014), T5(*P* = 0.006) and T6(*P* = 0.015) (Fig. 3).

Compared with the supine group, patients in the semi-Fowler’s position had significantly fewer severe coughing after extubation (Table 3, *P* = 0.008) and fewer episodes of immediate bucking after extubation (Table 3, *P*<0.001). Eight patients required suction after extubation in the semi-Fowler’s group, while 17 required it in the supine group (*P* = 0.052). Three of 141 patients vomited, including 2 semi-Fowler’s patients and 1 supine patient (*P* = 0.578). Four cases in each group experienced emergence agitation (*P* = 0.962), and the SAS scores were also comparable.

**Table 3:** Outcomes by Group

Outcome	Semi-fowler’s	Supine	*P* value
Sp0_2_ before extubation			
≥ 96%	70 (100%)	71 (100%)	
< 96%	0	0	
SpO2 decreased	0 (0%)	0 (0%)	
Suction(n(%))	8 (11.43%)	17 (23.94%)	0.052
Severe cough(n(%))	8 (11.43%)	21 (29.58%)	0.008
Bucking(n(%))			
Before extubation	9 (12.86%)	18 (25.35%)	0.059
Within 1 min after extubation	3 (4.29%)	18 (25.35%)	<0.001
Airway rescue(n(%))	1 (1.42%)	0 (0%)	0.323
Re-intubation(n(%))	0	0	
Vomiting(n(%))	2 (2.86%)	1 (1.41%)	0.578
Agitation(n(%))	4 (5.71%)	4 (5.63%)	0.962
SAS (mean ± SD)			
After posture placement	3.11 ± 1.16	3.35 ± 1.29	0.252
Before extubation	4.13 ± 0.48	4.21 ± 0.75	0.438
1 min after extubation	4.04 ± 0.43	4.06 ± 0.33	0.836

All *P*-values are calculated by Chi-square or Fischer’s exact test

One patient in the semi-Fowler’s position had a brief occurrence of upper airway obstruction, which required transient jaw-thrust maneuver. The use of dexmedetomidine and lidocaine cream was comparable between the two groups (Table 1).

All cases in the semi-Fowler’s position group received full satisfaction scores (4 points) from nursing personnel, while three cases in the supine position received 3 points and other three cases received 2 points (*P* = 0.013).

## Discussion

The safety and comfort associated with semi-Fowler’s position in emergence and extubation remain largely undocumented. This study demonstrated that the semi-Fowler’s position for emergence and extubation was a better choice for patients undergoing abdominal surgery than the traditional supine position.

In recent years, increasing attention has been paid to postural intervention in the ICU. Some studies have suggested that an early semi-supine position after intubation can reduce ventilator-associated pneumonia and be promoted in ICU [8, 10]. However, in PACU, postural intervention has rarely been reported. Some studies have found that in pediatric patients, extubation in a lateral position can reduce postoperative hypoxia [11]; others reported that decannulation in a prone position is safer in patients undergoing prone surgery [12].

In this study, the comfort VAS scores were significantly higher in the semi-Fowler’s position 5 min after extubation and when leaving PACU. The wound pain VAS scores were lower in the semi-Fowler’s position at all intervals after extubation, suggesting that the relief of wound pain by diminishing abdominal wall tension played a potential role in patient comfort in the semi-Fowler’s position. Conversely, the BCS scores based on the assessment of wound pain were similar between the two groups. The inconsistent results might be explained by the different standards for the evaluation and precision of these scales. These findings also implied that factors other than wound pain have an impact on the difference in patient comforts between the two groups. In the semi-Fowler’s position, the diaphragm moves downward, the work of breathing relatively decreases, lung volume and ventilation increases, and lung dilatation is promoted; these changes can improve oxygenation and increase oxygen saturation [13]. This advantage may improve patient comfort in PACU, especially in the stage of emergence from general anesthesia, when the incidence of residual neuromuscular blockage can be up to 64.7% [14]. The semi-Fowler’s position facilitates breathing of patients and may be one of the explanations for the higher comfort VAS scores in the semi-Fowler’s position group.

In the semi-Fowler’s position, changes in systemic circulatory blood volume might cause transient hypotension. Therefore, we paid careful attention to hemodynamic changes and cardiovascular safety. Our study found that, compared with basal blood pressure, there was no significant change in the MAP in both groups. However, there was a statistically significant difference in HR between the two groups. In the semi-Fowler’s position, the HR increased by 5 beats per minute; on the other hand, in the supine position, there was no significant change in HR. We speculate that this change was caused by the effect of posture on systemic circulatory blood volume, which was still within a safe range. In our study, all patients in both groups had normal baseline SpO_2_ before anesthesia, and oxygen saturation did not decrease significantly at any time points, remaining above 96%. Therefore, the semi-Fowler’s position did not show obvious advantages, which may be related to the continuous oxygen supply obtained by masking all patients and excluding patients with perioperative respiratory complications and smoking history during recruitment.

In the semi-Fowler’s position group, less severe coughing and passive bucking was observed. In surgical patients undergoing general anesthesia, both severe coughing and bucking can potentially result in dangerous complications, such as hypertension, tachycardia, and other arrhythmias, and have different adverse impacts on carotid endarterectomy, craniotomy, and ophthalmology [15, 16]. For abdominal surgery patients, severe coughing and bucking can induce an acute and violent rise in abdominal pressure, leading to obvious wound pain or wound dehiscence [17]. Our study revealed that extubation in the semi-Fowler’s position could significantly reduce bucking and severe coughing after extubation. However, effective expectoration needs moderate coughing. Single initiate coughing or coughing sustaining < 5 s was not recorded, but the cases requiring suction after extubation were less in the semi-Fowler’s position than in the supine position (11.43% vs. 23.94%, *P* = 0.052). Although expectoration is comparable between the two groups, the semi-Fowler’s position still provides better expectoration than the supine position without increasing unfavorable severe coughing and passive bucking. Some studies have shown that intravenous lidocaine or dexmedetomidine can reduce post-extubation coughing [18, 19]. In our study, there was no significant difference in the percentage of patients using lidocaine cream or intravenous dexmedetomidine.

In addition, considering that the semi-Fowler’s position may increase the workload of nursing, we also designed a satisfaction score for nurses. Interestingly, nurses gave a score of 4 to all cases in the semi-Fowler’s position, but 3 and 2 to three cases each in the other group (*P* = 0.013), which implied that nurses were more satisfied with the semi-Fowler’s position. One of the reasons explained by the nursing staff was that patients in the semi-Fowler’s position were more willing to handle their sputum excretion themselves, while those in the supine position were more likely to call for help. The other reason was that patients in the semi-Fowler’s position complained less for the pain or their discomfort. Contrary to our previous consideration, the semi-Fowler’s position decreased the workload of the nursing staff and achieved higher satisfaction from both patients and nurses.

This study has some limitations that should be addressed. First, it was conducted in a single hospital with a relatively inadequate sample. Second, the study includes a variety of surgical types, including laparoscopic colorectal cancer resection, upper gastrointestinal surgery, and urinary surgery, etc. And both laparoscopic and traditional open surgeries were involved. The size and position of surgical wounds have a potential impact on patients’ feelings and performance in the PACU. Further studies should be limited to a specific type of surgery, such as laparoscopic cholecystectomy, to minimize the bias. Third, in theory, semi-Fowler’s position is beneficial to respiratory recovery, but its advantages in respiration and oxygenation have not been found in this study. We plan to conduct further research in patients with a high risk of respiratory complications.

## Conclusion

In conclusion, this study demonstrated that the semi-Fowler’s position significantly relieved postoperative wound pain after abdominal surgery, reduced severe coughing and bucking after extubation, leading to better patient comfort and satisfaction from nursing staff, without increasing the risk of peri-extubation complications during PACU. Therefore, emergence and extubation in the semi-Fowler’s position should be considered as an alternative approach for patients undergoing abdominal surgery.

## Data Availability

All data used during this study are available from the corresponding author on reasonable request.

## Abbreviations

PACU: Post-anesthesia care unit
ASA: American Society of Anaesthesiologists
BMI: Body mass index
HR: Heart rate
MAP: Mean arterial pressure
VAS: Visual analog ue scale
SAS: Sedation-Agitation Scale
BCS: Bruggemann comfort scale
SD: Standard deviation
